# Association between Transforming Growth Factor-Beta 1 T869C Polymorphism and Ischemic Stroke: A Meta-Analysis

**DOI:** 10.1371/journal.pone.0067738

**Published:** 2013-07-05

**Authors:** Lingmei Peng, Peng Li, Jian Chen, Ke Yan, Fuyuan Huo, Lina Han, Can Li, Sheng Tan, Xiaodan Jiang

**Affiliations:** 1 The National Key Clinic Specialty, The Neurosurgery Institute of Guangdong Province, Guangdong Provincial Key Laboratory on Brain Function Repair and Regeneration, Department of Neurosurgery, Zhujiang Hospital, Southern Medical University, Guangzhou, China; 2 Department of Neurology, Zhujiang Hospital of Southern Medical University, Guangzhou, China; 3 Department of Neurology, The First People’s Hospital of Foshan, Foshan, China; 4 Department of Neurosurgery, The First People’s Hospital of Guangzhou, Guangzhou, China; University Medical Center Utrecht, The Netherlands

## Abstract

**Objective:**

To explore the association between transforming growth factor-beta1 (TGF-β1) T869C polymorphism and risk of ischemic stroke (IS) by performing a meta-analysis based on published articles.

**Methods:**

Systematic electronic searches of PubMed, Science Direct, BIOSIS Previews, Chinese Biomedical Database, Chinese National Knowledge Infrastructure, and WANFANG Database were performed. The strength of the association was calculated by pooled odds ratios (ORs) with 95% confidence intervals (95%CIs). Subgroup analysis was conducted to explore potential sources of heterogeneity. Sensitivity analysis was performed to elucidate the stability of the outcomes. Publication bias was evaluated by Begg’s funnel plot and Egger’s test.

**Results:**

A total of 6 studies involving 1701 cases were included. The overall estimates did not show any significant association between TGF-β1 T869C polymorphism and risk of IS under all genetic models (C vs. T: OR = 1.08,95%CI = 0.88–1.32; CC vs. TT:OR = 1.17,95%CI = 0.79–1.72; CT vs. TT: OR = 0.91, 95%CI = 0.68–1.22; CC+CT vs. TT: OR = 0.99, 95%CI = 0.73–1.35; CC vs. CT+TT: OR = 1.23, 95%CI = 0.95–1.59). Similar lacking associations were observed in subgroup analysis based on ethnicity and source of controls. When stratified by study design, significant increased association of IS risk was found in cohort studies under genetic models except recessive model(C vs. T: OR = 1.18, 95%CI = 1.05–1.32; CC vs. TT: OR = 1.40, 95%CI = 1.10–1.77; CT vs. TT: OR = 1.23, 95%CI = 1.02–1.49; CC+CT vs. TT: OR = 1.27, 95%CI = 1.03–1.57; CC vs. CT+TT, OR = 1.21, 95%CI = 0.99–1.47), whereas in case-control studies a significant decreased risk was detected under heterozygote comparison(CT vs. CC: OR = 0.72, 95%CI = 0.57–0.92). However, after correction for multiple testing, the associations were observed to be null significant in both cohort and case-control subgroups among all genetic models.

**Conclusion:**

This meta-analysis suggested that current epidemiological studies of TGF-β1 T869C polymorphism are too inconsistent to draw a conclusion on the association with IS susceptibility. Given the small sample size and remarkable between-study heterogeneity, further well-designed prospective large-scale studies are warranted.

## Introduction

Stroke is a leading cause of death and adult long-term disability worldwide, and remains as an enormous burden for society due to lack of illustrated etiology and effective treatments [1]. Approximately 87% of stroke cases are IS in origin. IS is a multifactorial disease involving complex interactions between genetic and environmental factors [2]. Emerging evidences have demonstrated that inflammation play an important role in the pathogenesis of IS even though the exact mechanism is still unknown [3]. As one of the candidate susceptible genes of IS, the functional polymorphism of inflammatory factors has become one of the most popular scientific focuses.

TGF-β1 is a pleiotropic cytokine with potent anti-inflammation property, and has been considered as an essential risk factor in the inflammatory process of IS by involving in the pathophysiological progresses of such as hypertension, atherosclerosis and lipid metabolisms [4]. The genetic polymorphisms of TGF-β1 can affect the level of its production.The TGF-β1 gene is located on chromosome 19(q13.1–13.3), including 7 exons and 6 introns. There are several common known single nucleotide polymorphisms (SNPs) in this gene. Among them, T869C (rs1982073; Leu10/Pro10; T29C, codon10) in exon 1 of the TGF-β1 gene is located at position 10 in the signal peptide; this sequence allows export of the newly synthesized protein across membranes of the endoplasmic reticulum. Different classes of signal sequence mutations changing one amino acid to another and affecting export efficiency have been described [5]. With a transition from T to C resulting in a substitution of leucine to proline, the T869C SNP was reported to be associated with elevated serum concentration of TGF-β1 [6]. Further functional experiments have been indicated that T869C polymorphism can increase the expression of TGF-β1 mRNA by influencing the intracellular trafficking or exporting efficiency of the synthesized protein to the endoplasmic reticulum, resulting in the elevated serum TGF-β1 level [7].

To date, several studies have been performed to investigate the association between T869C polymorphism of TGF-β1 gene and risk of IS. However, the results remain controversial [8], [9], [10], [11], [12], [13], [14]. Considering a single study may lack the power to provide reliable conclusion due to small amount of subjects and masses of statistical and clinical heterogeneities, we performed this meta-analysis combining eligible published literatures based on quantitative synthesis to derive a more convincing estimation for the association between T869C polymorphism and risk of IS.

## Materials and Methods

### Search Strategy

This meta-analysis was conducted in accordance with the Preferred Reporting Items for Systematic Reviews and Meta-analyses (PRISMA) criteria [15]. Systematic literature searches were performed through PubMed, Science Direct, BIOSIS Previews, Chinese Biomedical Database (http://sinomed.imicams.ac.cn/index.jsp), Chinese National Knowledge Infrastructure(http://dlib3.cnki.net/kns50/), and WANFANG Database (http://g.wanfangdata.com.cn/). Other relevant studies were also conducted by hand searching of the references of selected articles and the abstracts presented at related conferences. Languages were limited to English and Chinese. Search terms included the following key words: (“transforming growth factor beta-1″ OR “TGFβ1” OR “TGF-β1”) and (stroke OR “cerebral ischemia” OR “cerebral infarction” OR “brain infarction” OR “cerebrovascular accident”) and (“polymorphism OR mutation OR allele OR genotype OR variant OR variation”). If more than one geographic or ethnic group were included in one publication, each group was treated separately. The last search was updated on 10th March 2013.

### Selection Criteria

The inclusion criteria for identified studies were as follows: (1)evaluating the association between TGF-β1 T869C polymorphism and IS risk;(2)using a case-control, cohort, nested case-control, case-cohort, or cross-sectional study with either population-based or hospital-based design; (3)reporting sufficient available genotype distribution data in both cases and controls for calculating OR and 95%CI; (4) diagnosis of IS should be confirmed by computed tomographic(CT) or magnetic resonance imaging(MRI); and (5) genotype distribution in control group must be consistent with Hardy–Weinberg equilibrium (HWE). Studies were excluded if one of the following existed: (1) not relevant to TGF-β1 T869C polymorphism or IS; (2) genotype frequencies or numbers not reported; (3) the design based on family or sibling pairs; and (4) reviews, abstracts or animal studies. For studies with overlapped data by the same research group, the most recent or complete one was extracted. When detailed data were not reported, authors were contacted to obtain the relevant information.

### Data Extraction

Data were reviewed and extracted by 2 independent investigators (Peng and Li). Any disagreement was resolved by discussion and consensus with a third reviewer (Chen). The following data from eligible studies were extracted: the first author, publication year, country (ethnicity), study design, diagnostic criteria of IS, genotyping method, allele numbers and genotype frequencies in cases and controls.

### Quality Assessment

The quality of included studies was evaluated independently using Newcastle-Ottawa Scale (NOS) [16] based on the following three aspects: selection of cases and controls, comparability of cases and controls, and ascertainment of either the exposure or outcome of interest for case-control or cohort studies respectively. Studies with a score of 5 points or greater were considered to be of adequate quality.

### Statistical Analysis

Before this meta-analysis, power analysis was performed using the PS program(Power and Sample Size Calculation software, http://biostat.mc.vanderbilt.edu/twiki/bin/view/Main/PowerSampleSize) to prove whether the selected studies could offer adequate power. HWE of genotype distribution in the controls of eligible studies was calculated using an online program (http://ihg2.helmholtz-muenchen.de/cgi-bin/hw/hwa1.pl), and P value<0.05 suggested no deviation. The strength of association between TGF-β1 T869C polymorphism and IS risk was assessed by crude ORs with corresponding 95%CIs under the following five genetic models: the allele comparison(C vs. T), the homozygote comparison (CC vs. TT), the heterozygote comparison (CT vs. TT), the dominant model (CC+CT vs. TT), and the recessive model (CC vs. CT+TT). The significance of OR was determined by the Z-test, and P value<0.05 was accounted as statistically significant. The degree of heterogeneity between studies was detected by the Q-test and I^2^-statistics [17]. P_Q_ <0.10 or I^2^>50% suggested significant heterogeneity existed. The pooled estimate was combined by a random-effect model(DerSimonian-Laird method) [18] or a fixed-effect model (Mantel–Haenszel method) [19] according to whether the heterogeneity existed or not. Subgroup analysis based on ethnicity, source of controls, and study design were further performed respectively to explore the sources of heterogeneity. Bonferroni correction was utilized to adjust multiple testing. Because multiple comparisons in our meta-analysis were performed 35 times, the P value lesser than 0.05/35 (0.0014) was accepted for statistical significance after Bonferroni correction. Sensitivity analysis was conducted by subsequently omitting individual study to validate the reliability of the results. Publication bias was investigated by inspection of symmetry of Begg’s funnel plot and evaluation of Egger’s test [20], with a significant level set at P value less than 0.10. All analyses were conducted using software RevMan 5.1 and STATA 12.0.

## Results

### Study Characteristics

**Figure 1:**
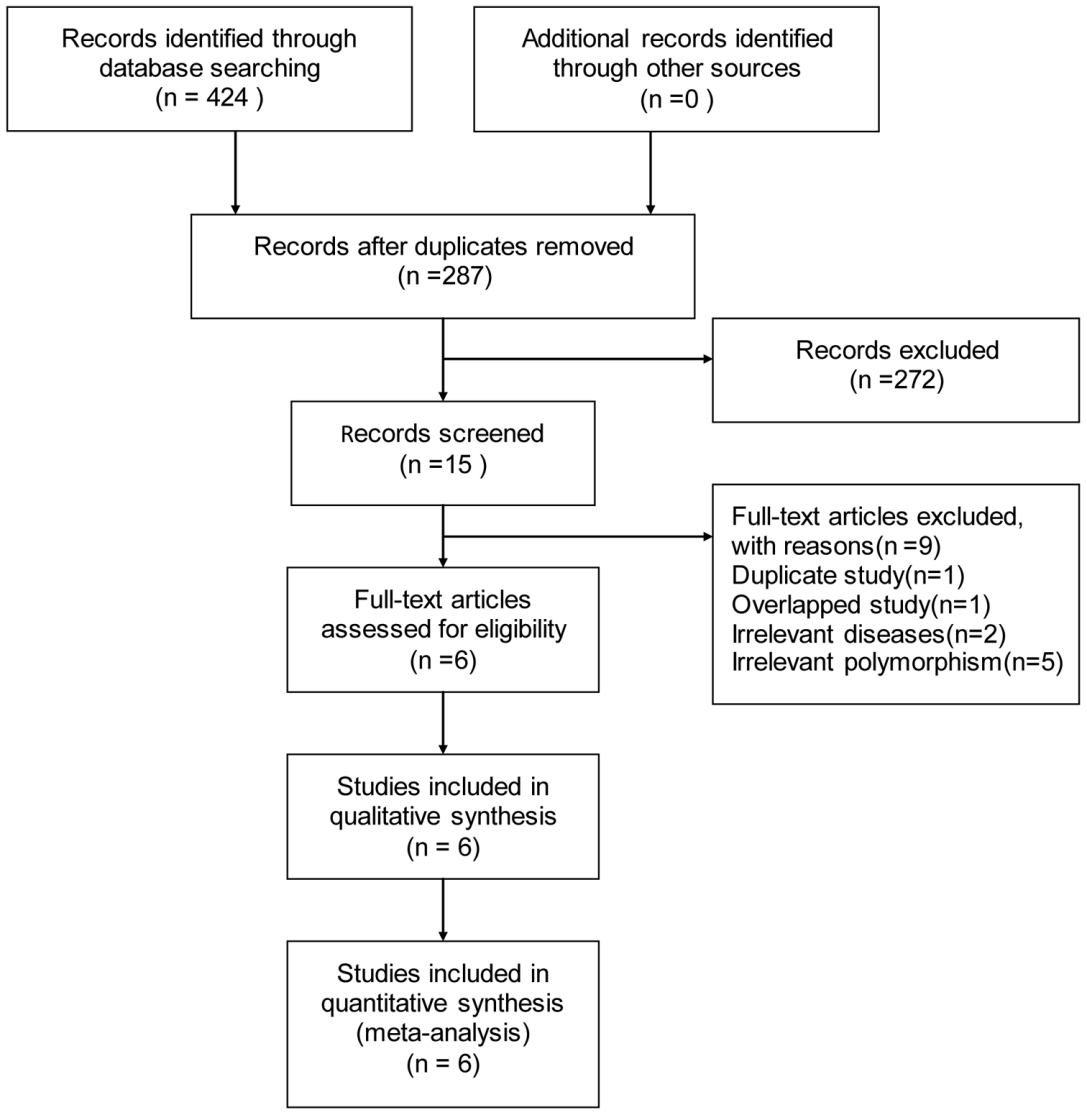
Flow diagram of the selection of eligible studies.

A flow chart summarizing selection process is detailed in Figure 1. The initial literature search identified 424 publications. Further selection of the inclusion criteria left 15 papers. Among these studies, 1 was duplicate study [21]; 2 investigated irrelevant diseases instead of IS [22], [23]; 4 focused on other polymorphisms of TGF-β1 gene [24], [25], [26], [27]; 2 involved overlapped data in case population and shared the same control population [11], , thus the one with more complete information was therefore included [11]. Finally, 6 studies involving 1701 cases were included in this meta-analysis [8], [9], [10], [11], [13], [28].

Among the 6 eligible studies, 4 were case-control studies in a population-based design [8], [9], [10], [11]; 1 was based on an ongoing population-based cohort study [13]; 1 was based on a cohort of type 2 diabetic patients [14]. The ethnicity of included subjects involved populations ranging from Caucasian (n = 1), Korean (n = 1), Japanese (n = 1) and Chinese (n = 3). As for the detailed definition of IS in cases, Kim et al [9] involved all subtypes of IS using TOAST classification, whereas Peng et al [8] only focused on atherosclerotic cerebral infarction; the remaining 4 studies investigated IS without further classification [10], [11], [13], [28].

The NOS score of each included studies was higher than 5 points, and the genotype distributions in all controls were consistent with HWE. The detailed characteristics of the eligible studies are listed in Table 1.

**Table 1:** Characteristics of studies included in the meta-analysis.

Study	Ethnicity	Study design	Control source	Genotype distribution(case/control)			HWE(P)	NOS
				TT	CT	CC		
Katakami2011	Japanese	Cohort	Hospital based	68/935	170/1689	93/838	0.17	6
Kim 2006	Korean	Case-control	Population based	79/42	123/110	69/55	0.33	7
Peng 2011	Chinese	Case-control	Population based	34/46	70/86	60/35	0.66	7
Sie 2006	Caucasian	Cohort	Population based	109/2348	142/2720	48/820	0.47	8
Tao 2010	Chinese	Case-control	Population based	152/111	193/217	105/122	0.46	8
Liang 2010	Chinese	Case-control	Population based	44/38	82/88	60/34	0.20	7

### Quantitative Synthesis

Overall, no significant association between TGF-β1 T869C polymorphism and risk of IS was observed under all genetic models (C vs. T: OR = 1.08,95%CI = 0.88–1.32; CC vs. TT:OR = 1.17,95%CI = 0.79–1.72; CT vs. TT: OR = 0.91, 95%CI = 0.68–1.22; CC+CT vs. TT: OR = 0.99, 95%CI = 0.73–1.35; CC vs. CT+TT: OR = 1.23, 95%CI = 0.95–1.59), with remarkable between-study heterogeneities (Figure S1); thus a random-effect model was employed to calculate the pooled estimates. The main results of pooled estimates in this meta-analysis are shown in Table 2.

**Table 2:** Summary risk estimates for association between TGF-β1 T869C polymorphism and IS.

Comparisons	Stratifications	Studies(n)	Pooled estimate		Heterogeneity	
			OR(95%CI) P_Z_		I^2^(%)	P_H_
C vs. T	Overall	6	1.08(0.88, 1.32)	0.77	81	<0.001
	Asians	5	1.08(0.83, 1.40)	0.58	85	<0.001
	Non-Asians	1	1.12(0.95, 1.33)	0.17	NA	NA
	Population-based	5	1.05(0.83, 1.34)	0.68	82	<0.001
	Hospital-based	1	1.23(1.05, 1.44)	0.01	NA	NA
	Case-control	4	1.04(0.75, 1.44)	0.19	85	0.001
	Cohort	2	1.18(1.05, 1.32)	0.005	0	0.450
CC vs. TT	Overall	6	1.17(0.79, 1.72)	0.44	79	<0.001
	Asians	5	1.15(0.70, 1.90)	0.58	83	<0.001
	Non-Asians	1	1.26(0.89, 1.79)	0.19	NA	NA
	Population-based	5	1.10(0.69, 1.75)	0.69	80	<0.001
	Hospital-based	1	0.90(0.76, 1.07)	0.01	NA	NA
	Case-control	4	1.07(0.58, 1.97)	0.83	83	<0.001
	Cohort	2	1.40(1.10, 1.77)	0.006	0	0.430
CT vs. TT	Overall	6	0.91(0.68, 1.22)	0.54	73	0.002
	Asians	5	0.87(0.60, 1.25)	0.44	76	0.002
	Non-Asians	1	1.12(0.87, 1.45)	0.37	NA	NA
	Population-based	5	0.83(0.62, 1.11)	0.20	63	0.030
	Hospital-based	1	1.38(1.03, 1.85)	0.03	NA	NA
	Case-control	4	0.72(0.57, 0.92)	0.008	17	0.310
	Cohort	2	1.23(1.01, 1.51)	0.04	9	0.290
CC+CT vs. TT	Overall	6	0.99(0.73, 1.35)	0.96	79	<0.001
	Asians	5	0.96(0.64, 1.43)	0.83	82	<0.001
	Non-Asians	1	1.16(0.91, 1.47)	0.24	NA	NA
	Population-based	5	0.91(0.66, 1.26)	0.59	75	0.003
	Hospital-based	1	1.43(1.08, 1.89)	0.01	NA	NA
	Case-control	4	0.84(0.58, 1.23)	0.37	69	0.040
	Cohort	2	1.27(1.03, 1.57)	0.02	23	0.020
CC vs. CT+TT	Overall	6	1.23(0.95, 1.59)	0.12	68	0.009
	Asians	5	1.25(0.90, 1.73)	0.18	78	0.004
	Non-Asians	1	1.18(0.86, 1.62)	0.30	NA	NA
	Population-based	5	1.24(0.88, 1.75)	0.21	74	0.004
	Hospital-based	1	1.22(0.95, 1.57)	0.12	NA	NA
	Case-control	4	1.28(0.80, 2.04)	0.30	80	0.002
	Cohort	2	1.21(0.99, 1.47)	0.26	0	0.87

NA, data not available; P_Z,_ P value for Z test; P_H_, P value for heterogeneity.

**Figure 2:**
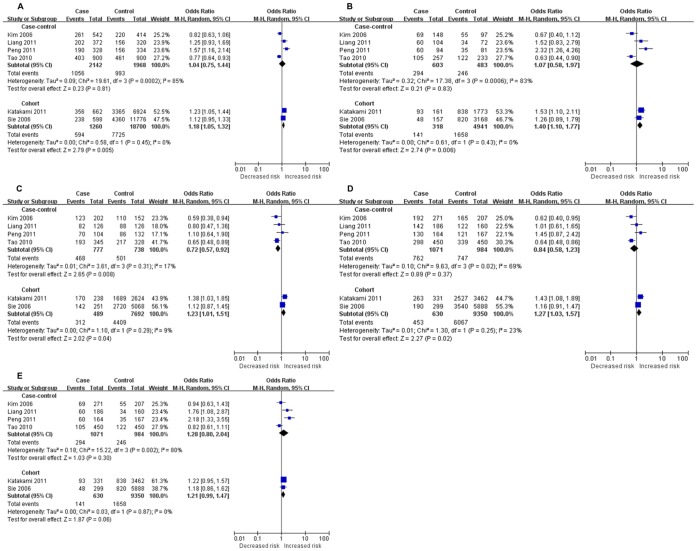
Forest plot for association between TGF-β1 T869C polymorphism and IS risk based on study design. (A) Allele comparison(C vs. T); (B) Homozygote comparison (CC vs. TT); (C) Heterozygote comparison (CT vs. TT); (D) Dominant model (CC+CT vs. TT); (E) Recessive model (CC vs. CT+TT).

To explore the sources of heterogeneity, further subgroup analyses by ethnicity, source of controls, and study design were performed respectively. Similarly, lacking significant associations were revealed in the subgroup of Asians and population-based studies, yet still with significant heterogeneity among all contrasts. As for non-Asians or hospital-based studies, we were unable to draw a conclusion since both of the two stratifications included only one study(Table 2). When stratified by study design, significant association of increased IS risk was observed in cohort studies under allele comparison, homozygote comparison, heterozygote comparison, and dominant model (C vs. T: OR = 1.18, 95%CI = 1.05–1.32; CC vs. TT: OR = 1.40, 95%CI = 1.10–1.77; CT vs. TT: OR = 1.23, 95%CI = 1.02–1.49; CC+CT vs. TT: OR = 1.27, 95%CI = 1.03–1.57; CC vs. CT+TT, OR = 1.21, 95%CI = 0.99–1.47), whereas in control studies we found a decreased risk under heterozygote comparison(CT vs. CC: OR = 0.72, 95%CI = 0.57–0.92)(Figure 2). However, after Bonferronic correction for multiple testing, the associations were observed to be null significant in both cohort and case-control subgroups among all genetic models. After stratifying by study design, between-study heterogeneity was remarkable decreased in cohort studies, yet still detectable in case-control studies (Table 2 and Figure 2). Power calculation on the pooled sample size showed that the statistical power was 54.5%, which was lower than 80%.

### Sensitivity Analysis

Sensitivity analysis was performed to examine the stability of the results. The corresponding pooled ORs were not significantly altered after excluding each eligible study at a time (data not shown).

### Publication Bias

The shape of the Begg’s funnel plot showed no asymmetric visually (figure not shown), and the result of Egger’s test detected no statistical evidence of publication bias among studies (P = 0.91 for allele comparison; P = 0.35 for homozygote comparison; P = 0.24 for heterozygote comparison; P = 0.48 for dominant model; P = 0.32 for recessive model) (Table 3).

**Table 3:** Publication bias tests for association between TGF-β1 T869C polymorphism and IS.

Comparisons	Egger test			Begg test
	Coefficient	P value	95%CI	P value
C vs. T	0.53	0.97	(−0.63,0.65)	0.91
CC vs. TT	4.32	0.35	(−6.92,15.57)	0.35
CT vs. TT	−3.93	0.24	(−11.82,3.94)	0.24
CC+CT vs. TT	−2.85	0.48	(−13.06,7.36)	0.48
CC vs. CT+TT	3.15	0.32	(−4.50,10.79)	0.32

## Discussion

It is well-established that inflammation and IS are interrelated. TGF-β1 is a multifactorial inflammatory cytokine involving in many inflammation processes as well as the development of IS. The expression of plasma TGF-β1 protein is predominantly under genetic control and modified by several common SNPs in this gene, and T869C polymorphism has been indicated to be associated with elevated serum TGF-β1 level. Several epidemiological studies investigated the association between T869C polymorphism and susceptibility to IS, yet with conflicting results. Therefore, we conducted this meta-analysis combining all eligible published studies to draw a more precise conclusion.

Our overall estimates did not observed any significant association between TGF-β1 T869C polymorphism and risk of IS under all contrasts, as well as in subgroup analysis by ethnicity or source of controls. However, since there was only one study included in non-Asian and hospital-based group, these results may not be valid. In subgroup analysis based on study design, significant but contradictory results were found between case-control and cohort studies. Likewise, since there were only two studies included in cohort group, and one of which was hospital-based source in controls, we may run the risk of chance bias. Moreover, after correction for multiple testing, the associations were observed to be null significant in both cohort and case-control subgroups among all genetic models. Due to these discrepancies, we were unable to draw a solid conclusion on the association between T869C polymorphism and the risk of IS.

Obvious evidences of between-study heterogeneities existed among all comparison models. Although the heterogeneity was almost removed when stratified by study design, remarkable heterogeneity still existed after stratification based on ethnicity or source of controls, suggesting that all above confounders should be taken into consideration. In addition, other factors such as gender distribution, subtype of IS, genotyping methods, as well as clinical characteristics such as smoking, alcohol drinking, family history of stroke, diabetes mellitus, hypertension, dyslipidemia, cardiac diseases and so on, might also account for the potential sources of heterogeneity. However, we failed to perform further subgroup analysis or meta-regression analysis based on these factors owing to limited information of original studies.

To our knowledge, the present study is the first meta-analysis concerning relationship between TGFβ1 gene T869C polymorphism and susceptibility to IS. Genotype distribution in all controls did not deviate from HWE, reassuring the representation of control samples. The result of NOS indicated that the methodological quality we adopted was of credibility. Moreover, sensitivity analysis did not significantly alter in overall and subgroup results under all genetic models; In addition, results from Begg’s funnel plot and Egger’s test indicated no evidence of publication bias. In addition, we conducted multiple testing to adjust for multiple comparisons which can reduce the type I error rate. Taken together, the outcomes we obtained were relatively reliable and stable.

Nevertheless, several limitations of the current study should be acknowledged. Firstly, the sample size of included studies was respectively small, attributing to a smaller amount for further subgroup analysis. Although meta-analysis has the benefit to overcome this limitation and may generate more precise results, the combined sample sizes in our study were still inadequate to detect the association between T869C polymorphism and IS risk because power calculation for the pooled sample sizes was less than 80%, which might attenuate the statistical power to detect a slight effect and increase the chance of opportunity bias.

Secondly, significant heterogeneity was observed among all genetic models. However, lacking detailed information of individual studies limited our further subgroup analysis or meta-regression to adjust potential confounders, which may result in obscure estimates.

Thirdly, although we performed a systematic searching strategy to identify eligible studies, there was still probability that few studies so called “grey literatures” were not included. In addition, since we restricted literatures published in English or Chinese language, potential eligible studies written in other languages would also be neglected.

Fourthly, our current study only focused on one variation in TGF-β1 gene, without considering the role of other SNPs in this gene nor precluding the potential interactions such as linkage disequilibrium or haplotype analysis between them, which might mask its real genetic effect towards IS susceptibility.

Last but not the least, IS is a complex multifactorial disease related to both genetic and environmental factors, such as climate, diet, life style and economic status, etc. However, our results were based on unadjusted estimates since most of original studies did not take such gene-environmental interactions into account.

In summary, this meta-analysis suggested that current epidemiological studies of TGF-β1 T869C polymorphism are too inconsistent to draw a conclusion on the association with IS susceptibility. However, given the limitations above, more well-designed prospective large-scale studies with different ethnicities considering both genetic and environmental factors are warranted in future.

## Supporting Information

Figure S1 **Forest plot of all genetic models for association between TGF-β1 T869C polymorphism and IS risk.**
(TIF)

Checklist S1 **PRISMA 2009 Checklist.**
(DOC)
