# Supplementary figures and images for: Association between Transforming Growth Factor-Beta 1 T869C Polymorphism and Ischemic Stroke: A Meta-Analysis
Source: PLoS One. 2013 Jul 5;8(7):e67738. doi: 10.1371/journal.pone.0067738 (PMC3702507; doi:10.1371/journal.pone.0067738)

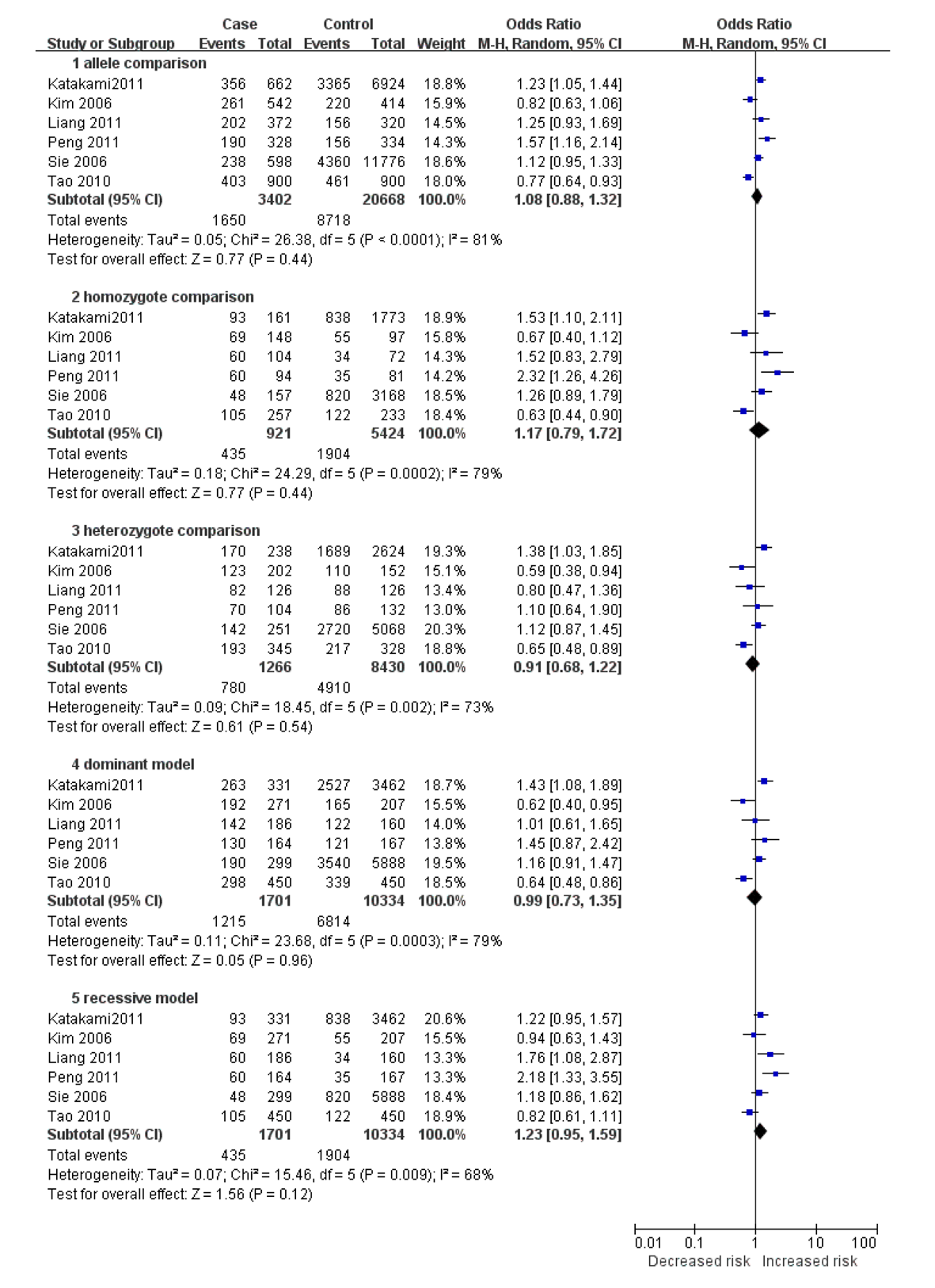

Supplement: Figure S1 — Forest plot of all genetic models for association between TGF-β1 T869C polymorphism and IS risk. (TIF) [file pone.0067738.s001.tif]
